# Heart fatty acid binding protein and Aβ-associated Alzheimer’s neurodegeneration

**DOI:** 10.1186/1750-1326-8-39

**Published:** 2013-10-02

**Authors:** Rahul S Desikan, Wesley K Thompson, Dominic Holland, Christopher P Hess, James B Brewer, Henrik Zetterberg, Kaj Blennow, Ole A Andreassen, Linda K McEvoy, Bradley T Hyman, Anders M Dale

**Affiliations:** 1 Department of Radiology, University of California, San Diego, 8950 Villa La Jolla Drive, Suite C101, La Jolla, CA 92037-0841, USA; 2 Department of Psychiatry, University of California, San Diego, La Jolla, CA, USA; 3 Department of Neurosciences, University of California, San Diego, La Jolla, CA, USA; 4 Neuroradiology Section, Department of Radiology and Biomedical Imaging, University of California, San Francisco, CA, USA; 5 Clinical Neurochemistry Laboratory, The Sahlgrenska Academy at Göteburg University, Gotheburg, Mölndal 40530, Sweden; 6 UCL Institute of Neurology, Queen Square, London WC1N 3BG, UK; 7 Institute of Clinical Medicine, University of Oslo and Division of Mental Health and Addiction, Oslo University Hospital, Oslo, Norway; 8 Department of Neurology, Massachusetts General Hospital, Boston, MA, USA

**Keywords:** Alzheimer’s disease, Fatty acids, Lipids, Amyloid, Tau, Brain atrophy

## Abstract

**Background:**

Epidemiological and molecular findings suggest a relationship between Alzheimer’s disease (AD) and dyslipidemia, although the nature of this association is not well understood.

**Results:**

Using linear mixed effects models, we investigated the relationship between CSF levels of heart fatty acid binding protein (HFABP), a lipid binding protein involved with fatty acid metabolism and lipid transport, amyloid-β (Aβ), phospho-tau, and longitudinal MRI-based measures of brain atrophy among 295 non-demented and demented older individuals. Across all participants, we found a significant association of CSF HFABP with longitudinal atrophy of the entorhinal cortex and other AD-vulnerable neuroanatomic regions. However, we found that the relationship between CSF HABP and brain atrophy was significant only among those with low CSF Aβ_1–42_ and occurred irrespective of phospho-tau_181p_ status.

**Conclusions:**

Our findings indicate that Aβ-associated volume loss occurs in the presence of elevated HFABP irrespective of phospho-tau. This implicates a potentially important role for fatty acid binding proteins in Alzheimer’s disease neurodegeneration.

## Background

A growing number of epidemiological and experimental studies suggest an association between Alzheimer’s disease (AD) and dyslipidemia. In observational studies, high serum cholesterol levels have been associated with increased risk of AD [1,2]. Genetic linkage and genome-wide association studies have identified a number of genes involved with cholesterol metabolism and transport as AD susceptibility loci [3,4] and cellular and molecular biology research has indicated a critical role for neuronal membrane phospholipids ('lipid rafts’) in modulating AD-associated pathogenesis [5]. However, in animal models, apolipoprotein E modulates the relationship between low-density lipoproteins and amyloid-β (Aβ) deposition [6,7] suggesting an indirect effect of intra-cranial cholesterol on Alzheimer’s pathology. Furthermore, conflicting evidence from epidemiological studies shows an unclear association between elevated cholesterol levels and AD risk [8,9], protein levels of fatty acid binding proteins have been noted to be decreased in brains of AD patients [10], and randomized clinical trials have not shown a clear benefit of lipid-lowering therapy on AD onset [2]. Thus there is a need for additional research evaluating the relationship between lipid biology and neurodegeneration in individuals at risk for AD.

In humans, structural MRI and CSF biomarkers allow for the indirect assessment of the cellular changes underlying AD *in vivo*. Structural MRI provides measures of brain atrophy, which reflect loss of dendrites, synapses [11] and neurons [12]. Low CSF levels of Aβ strongly correlate with intracranial amyloid plaques and high concentrations of CSF phospho-tau (p-tau) correlate with tau-associated neurofibrillary tangles [13,14]. Recent work suggests that CSF levels of heart fatty acid binding protein (HFABP or FABP3), a lipid binding protein involved with fatty acid metabolism and lipid transport [15] may have diagnostic and prognostic value in the earliest stages of AD [16-20]. Here, among non-demented older individuals at risk for AD and demented participants diagnosed with probable AD, we investigated whether CSF HFABP is associated with brain atrophy over time and whether interactions between high CSF HFABP and low CSF Aβ_1–42_ and high CSF HFABP and high CSF p-tau_181p_ are associated with brain atrophy over time. We also evaluated the relationship between CSF HFABP and other lipid binding proteins including Apolipoprotein (Apo) C III, Apo D, and Apo E.

## Results

### CSF HFABP and brain atrophy rates

**Figure 1:**
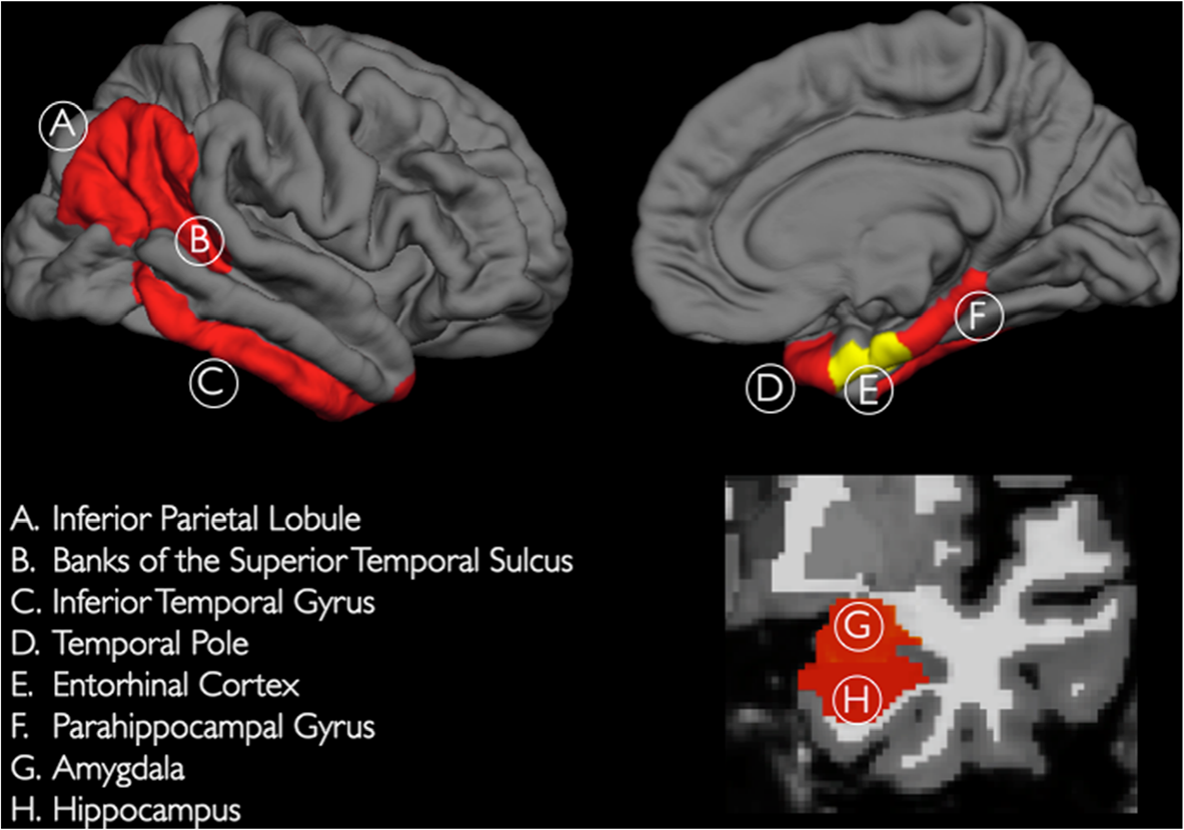
**Three-dimensional representations of the neuroanatomic regions examined in the current study (only one hemisphere is shown).** All of the examined neocortical regions are illustrated in the lateral and medial views of the gray matter surface (top row). The two non-neocortical regions (i.e., the hippocampus and amygdala) are illustrated in the coronal view of a T1-weighted MRI image (bottom row). Regions illustrated in red constitute the 'AD-vulnerable ROI’ (for further details please see manuscript text).

In our initial analyses, we used linear mixed effects models, co-varying for baseline age, sex, presence ("carriers") or absence ("non-carriers") of at least one ϵ4 allele of apolipoprotein E (APOE ϵ4), diagnostic status (AD vs mild cognitive impairment (MCI) vs healthy elderly controls (HC)), and disease severity (CDR-Sum of Boxes score at baseline) to examine whether CSF HFABP levels are associated with longitudinal atrophy of the entorhinal cortex and other AD-vulnerable regions of interest ('AD vulnerable ROI’ – for additional details see Methods) (Figure 1). We found a significant relationship between CSF HFABP, time, and atrophy rate of the entorhinal cortex (β-coefficient = -0.007, standard error (SE) = 0.003, p-value = 0.013) and the AD-vulnerable ROI (β-coefficient = -0.005, SE = 0.002, p-value = 0.017), indicating increased volume loss with elevated CSF HFABP levels.

### CSF HFABP, CSF Aβ_1–42_, CSF p-tau_181p_, and brain atrophy rates

Next, we asked whether statistical interactions between CSF HFABP and CSF Aβ_1–42_ and between CSF HFABP and CSF p-tau_181p_ are associated with brain atrophy over time. These linear mixed effects models included the main and interactive effects of CSF HFABP, CSF Aβ_1–42,_ status and CSF p-tau_181_ status, and co-varied for the demographic and clinical variables mentioned previously (see Methods for further details on the model). Key results from these models are shown in Table 1.

**Table 1:** Linear mixed effects model results for analyses involving all 295 participants

	**Main effect**	**Interaction with time**	**Interaction with HFABP and time**
***Entorhinal Cortex***			
CSF HFABP	0.005 (0.15)	-0.005 (0.34)	N/A
CSF Aβ_1–42_ status	0.003 (0.17)	-0.0004 (0.88)	-0.017 (0.003)
CSF p-tau_181p_ status	-0.004 (0.15)	-0.01 (0.0007)	0.020 (0.0005)
***AD-Vulnerable ROI***			
CSF HFABP	0.003 (0.21)	-0.003 (0.51)	N/A
CSF Aβ_1–42_ status	0.001 (0.44)	-0.0000 (0.99)	-0.013 (0.002)
CSF p-tau_181p_ status	-0.002 (0.10)	-0.0007 (0.002)	0.014 (0.001)
		**Time by HFABP Entorhinal cortex**	**Time by HFABP AD Vulnerable ROI**
Low CSF Aβ_1–42_ (n = 211)		-0.009 (0.007)	-0.007 (0.007)
High CSF Aβ_1–42_ (n = 86)		0.001 (0.75)	0.003 (0.22)
High CSF p-tau_181p_ (n = 197)		-0.002 (0.58)	-0.002 (0.47)
Low CSF p-tau_181p_ (n = 98)		-0.006 (0.21)	-0.002 (0.35)

Beta-coefficients with p-values indicated in parenthesis. Please see text for additional details.

As illustrated in Table 1, with the interaction terms of the three CSF biomarkers in the model, the association of CSF HFABP with brain atrophy over time was not significant. However, there were significant interactive effects between CSF HFABP x CSF Aβ_1–42_ status x time and CSF HFABP x CSF p-tau_181p_ status x time on atrophy of both the entorhinal cortex and the AD-vulnerable ROI. The only CSF biomarker to show a significant association with atrophy over time in the entorhinal cortex and AD-vulnerable ROI was CSF p-tau_181p_. Of the co-variates, the interaction of time by diagnostic status and APOE ϵ4 carrier status were significant for both entorhinal cortex atrophy (diagnostic status x time: β-coefficient = -0.007, SE = 0.002, p-value = 0.0005; APOE ϵ4 carrier status x time (β-coefficient = -0.004, SE = 0.002, p-value = 0.03) and AD-vulnerable ROI atrophy (diagnostic status x time: β-coefficient = -0.006, SE = 0.002, p-value = 0.0003; APOE ϵ4 carrier status x time (β-coefficient = -0.003, SE = 0.001, p-value = 0.02). None of the other variables showed significant main or interactive effects.

**Figure 2:**
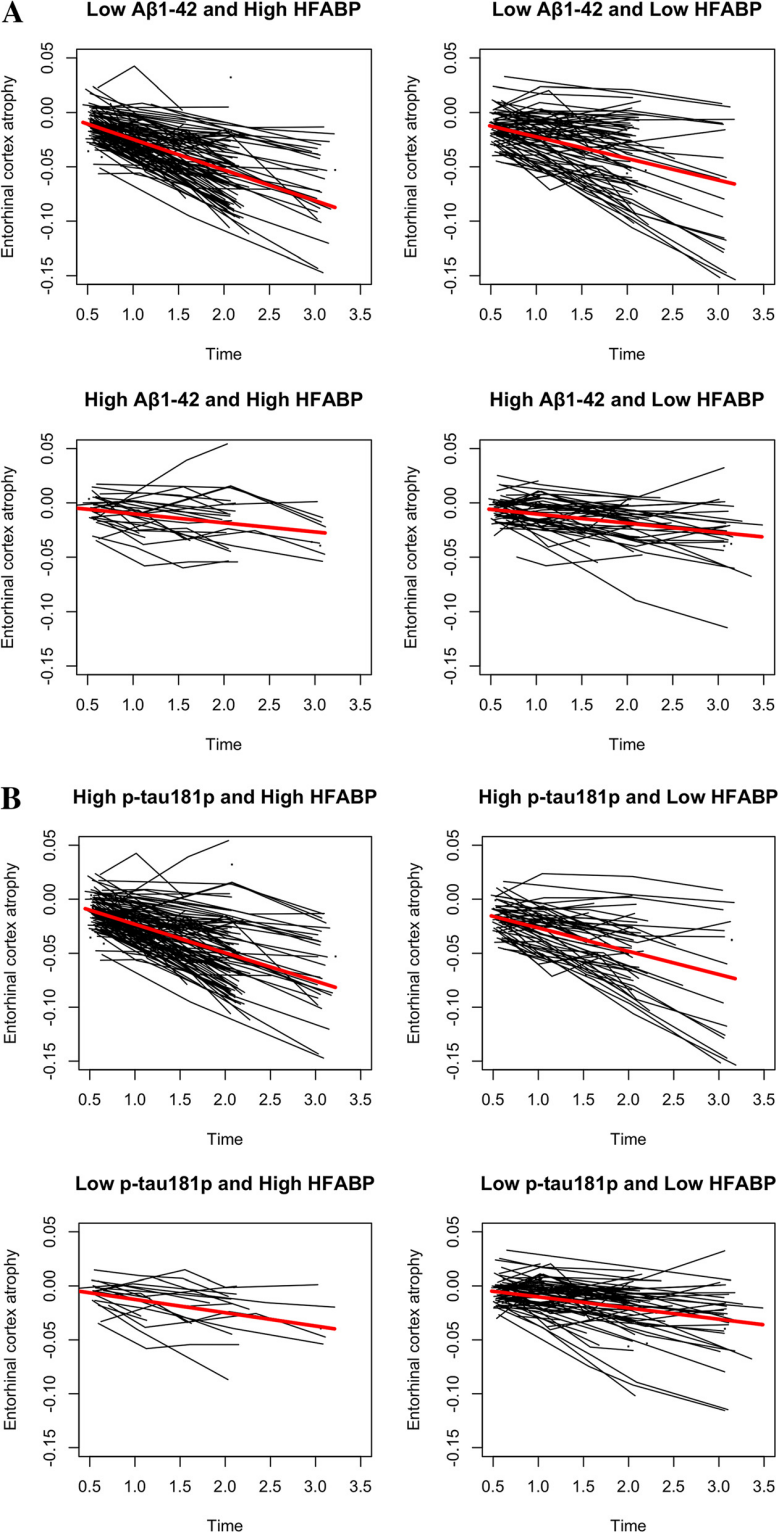
**(A) Spaghetti plots illustrating atrophy of the entorhinal cortex among all participants classified as low Aβ**_**1–42 **_**and high HFABP (based on median value of FABP) (top left panel), low Aβ**_**1–42 **_**and low HFABP (top right panel), high Aβ**_**1–42 **_**and high FABP (bottom left panel), and high Aβ**_**1–42 **_**and low FABP (bottom right panel).** The red line indicates the mean atrophy rate for the four respective groups (i.e. low Aβ_1–42_ and high FABP, low Aβ_1–42_ and low FABP, high Aβ_1–42_ and high FABP and high Aβ_1–42_ and low FABP). As illustrated, the slopes of the red lines are significantly different depending on CSF Aβ_1–42_ status (please see text for further details). **(B)** Spaghetti plots illustrating atrophy of the entorhinal cortex among all participants classified as high p-tau_181p_ and high HFABP (based on median value of FABP) (top left panel), high p-tau_181p_ and low HFABP (top right panel), low p-tau_181p_ and high FABP (bottom left panel), and low p-tau_181p_ and low FABP (bottom right panel). The red line indicates the mean atrophy rate for the four respective groups (i.e. high p-tau_181p_ and high FABP, high p-tau_181p_ and low FABP, low p-tau_181p_ and high FABP and low p-tau_181p_ and low FABP). As illustrated, the slopes of the red lines are not significantly different depending on CSF p-tau_181p_ status (please see text for further details).

To further investigate the three-way interactions, we performed follow-up analyses after stratifying on the basis of CSF Aβ_1–42_ status (i.e. low and high status) and CSF p-tau_181p_ status (i.e. high and low status). We found a significant CSF HFABP by time interaction on entorhinal cortex and AD-vulnerable ROI atrophy rate only among individuals with low CSF Aβ_1–42_ levels (Table 1, Figure 2A) Among individuals with high CSF Aβ_1–42_ levels and high or low CSF p-tau_181p_ levels, we found no effect of CSF HFABP by time on brain atrophy (Table 1, Figure 2A, 2B). This indicates elevated volume loss with elevated CSF HFABP and low CSF Aβ_1–42_ irrespective of CSF p-tau_181p_ status.

We also evaluated whether interactions between high CSF HFABP and low CSF Aβ_1–42_ and high CSF HFABP and high CSF p-tau_181p_ are associated with longitudinal clinical decline as assessed with the Alzheimer’s Disease Assessment Scale-cognitive subscale (ADAS-cog). In these analyses, neither the interaction between CSF HFABP, CSF Aβ_1–42_ status and time nor the interaction between CSF HFABP, CSF p-tau_181p_ status and time was significantly associated with longitudinal change in ADAS-cog (see Additional file 1).

### CSF HFABP and other lipid binding proteins

We next used generalized linear models to investigate the relationship between CSF HFABP and CSF p-tau_181p_, and levels of other lipid binding proteins including Apolipoprotein (Apo) C III, Apo D, and Apo E at baseline. The relation of each of these apolipoproteins with CSF HFABP was assessed in separate models; all models controlled for age, sex, APOE ϵ4 carrier status, diagnostic status, and CDR-Sum of Boxes score.

**Figure 3:**
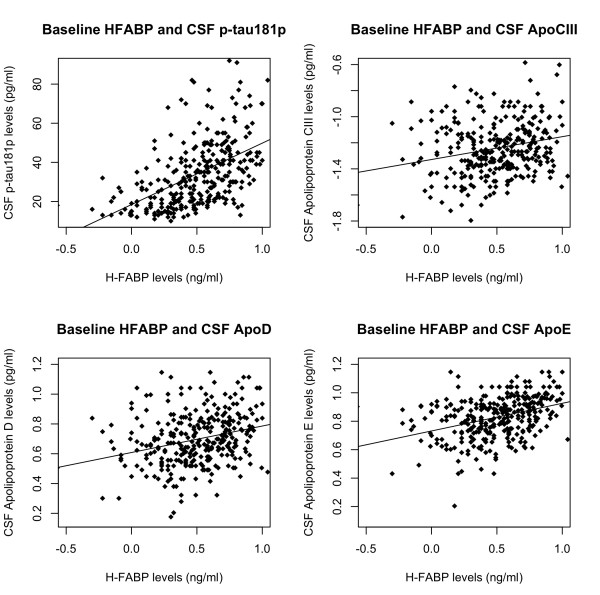
**Scatter plots demonstrating the relationship between baseline CSF levels of HFABP (quality-controlled, transformed values as described in reference 18) CSF p-tau**_**181p **_**(top left), CSF ApoC III (top right), CSF ApoD (bottom left) and CSF ApoE (bottom right).** The black line represents the best-fit regression line.

We found significant associations between CSF HFABP and CSF p-tau_181p_ (β-coefficient = 0.008, SE = 0.001, p-value = < 2.0 x 10^-16^), CSF levels of Apo C III (β-coefficient = 0.29, SE = 0.07, p-value = 4.2 x 10^-5^), ApoD (β-coefficient = 0.35, SE = 0.09, p-value = 2.8 x 10^-4^), and ApoE (β-coefficient = 0.86, SE = 0.12, p-value = 2.9 x 10^-16^) (Figure 3).

Using the same linear mixed effects framework described above, we additionally evaluated whether statistical interactions between CSF HFABP and the other lipid binding proteins are associated with brain atrophy over time. We found a significant interaction only between CSF HFABP and CSF Apo C III on entorhinal cortex atrophy rate (β-coefficient = 0.02, SE = 0.009, p-value = 0.02). None of the other lipid binding proteins demonstrated a significant interaction with CSF HFABP on atrophy rates of the entorhinal cortex and the AD-vulnerable ROI (p-values > 0.2).

## Discussion

Here, we demonstrate that in non-demented older individuals at risk for AD and in mild AD participants, HFABP is associated with volume loss in brain areas selectively affected in the earliest stages of AD. However, we found that the relationship between HFABP and brain atrophy is present only among individuals with low CSF Aβ_1–42_, and occurs irrespective of phospho-tau levels. Considered together, these results suggest a potentially important role for fatty acid binding proteins in early Alzheimer’s disease pathobiology.

Our findings also indicate that rather than simply representing a generalized marker of neuronal degeneration, elevated CSF levels of HFABP may reflect central nervous system lipid dyshomeostasis. Consistent with prior studies [16,18], we found a significant association between HFABP and phospho-tau. However, we also found a strong relationship between HFABP and a number of apolipoproteins including ApoE, as well as an interaction between HFABP and ApoC III on volume loss. Together, these findings support the hypothesis that intra-cranial lipid biology may influence Alzheimer’s neurodegeneration [5].

Our results suggest that the relationship between neuronal lipid biology and neurodegeneration may be influenced by amyloid pathology even after controlling for the effects of APOE ϵ4. An important aspect of our findings is the specific relationship between HFABP and Aβ deposition, where volume loss occurs only in the presence of elevated HFABP and decreased Aβ (i.e. increased Aβ deposition in the brain). A growing body of experimental evidence from various model systems indicates that phospholipids play an integral role in regulating amyloidogenesis. Enriched cholesterol and lipid microenvironments ('lipid rafts’) within the plasma membrane and the mitochondria-associated endoplasmic reticulum membrane promote γ-secretase activity resulting in increased Aβ production [5,21]. In comparison, outside lipid rafts, amyloid-precursor protein (APP) processing occurs predominantly via the non-amyloidogenic α-secretase pathway [22]. Membrane phospholipids also influence Aβ aggregation and clearance [5] and through release of arachidonic acid via the phospholipase 2 pathway, may additionally serve as critical mediators in Aβ-induced synaptoxicity, leading to learning, memory, and behavioral impairments in mouse models of AD [23]. Our results are consistent with these observations and suggest that phospholipids and lipid binding proteins may affect Alzheimer’s neurodegeneration primarily via Aβ-associated mechanisms.

One potential concern is that our current findings do not explain the previously noted relationships observed between HFABP and non-AD neurodegenerative diseases such as vascular dementia, Creutzfeldt-Jakob disease, Parkinson’s disease, and dementia with Lewy bodies [18,24-27]. Though we did not explicitly evaluate the role of HFABP in other neurodegenerative conditions or as a differential disease marker of AD, our findings may help provide insights into common mechanisms underlying an array of protein misfolding neurologic disorders. For example, lipid metabolism may play an important role in synaptic degeneration and regeneration [28] and as such, may be involved in a number of neurodegenerative diseases. Another intriguing notion is that in addition to APP and Aβ, phospholipid rich lipid rafts may play an important role in mediating pathogenesis associated with a number of proteins including α-synuclein [29,30] and prions [31,32] thus raising the possibility that lipid dyshomeostasis may represent an early trigger for a number of protein misfolding neurodegenerative disorders.

A limitation of our study is its observational nature, which precludes conclusions regarding causation. Our results cannot differentiate whether elevated HFABP causes, results from, or is simply correlated with amyloid deposition and volume loss. Additionally, our findings require further validation on a larger, independent population-based cohort.

## Conclusions

In summary, our results suggest that CSF HFABP reflects intra-cranial lipid biology and associates with Aβ-associated neurodegeneration irrespective of tau. Clinically, our findings suggest that HFABP may represent an important modifier of progression from amyloid deposition to neurodegeneration. Considered together with our prior findings [33,34], this suggests that in addition to phospho-tau, the HFABP/Aβ/neurodegeneration axis may represent an important area for further investigation. Using experimental models, it would be helpful to better understand the precise relationship between HFABP and Aβ dyshomeostasis and whether proteins influence or modulate this association. The results of these studies could provide insights into whether fatty acids and lipids represent a viable therapeutic target for individuals in the presymptomatic and very mildly symptomatic phase of the disease process.

## Methods

We evaluated cognitively normal older adults (HC = 90), individuals diagnosed with amnestic MCI (n = 139), and probable AD (n = 66) from the Alzheimer’s Disease Neuroimaging Initiative (ADNI; see Additional file 1 for further details). From a total of 415 demented and non-demented older individuals who underwent longitudinal MR imaging and CSF lumbar puncture, we restricted our analyses to those participants with quality-assured baseline and at least one follow-up MRI scan (Table 2).

**Table 2:** Demographic, clinical, and imaging data for all participants in this study

	**HC (n = 90)**	**MCI (n = 139)**	**AD (n = 66)**
Age, Mean (SE)	76.0 (0.6)	75.1 (0.7)	75.4 (0.9)
Female, %	51	33	41
Education Years, Mean (SE)	15.6 (0.3)	16.1 (0.2)	15.1 (0.4)
MMSE, Mean (SE)	29.1 (0.1)	26.7 (0.1)	23.4 (0.2)
CDR-SB, Mean (SE)	0.03 (0.01)	1.5 (0.07)	4.2 (0.2)
APOE ϵ4 carriers (%)	24	54	71
CSF H-FABP level (ng/ml), Mean (SE)	0.38 (0.03)	0.54 (0.02)	0.58 (0.03)
CSF Aβ_1–42_ level (pg/ml), Mean (SE)	207.8 (5.6)	157.5 (4.1)	141.5 (2.6)
CSF p-tau_181p_ level (pg/ml), Mean (SE)	24.7 (1.4)	36.8 (1.3)	41.7 (2.6)
Entorhinal Cortex APC, Mean (SE)	-0.8 (0.1)	-2.4 (0.1)	-2.9 (0.2)
AD-vulnerable ROI APC, Mean (SE)	-1.1 (0.1)	-3.2 (0.2)	-3.9 (0.3)

*MCI*; mild cognitive impairment, *HC*; cognitively normal older adults, *AD*; Alzheimer’s disease, *MMSE*; Mini-mental status exam, *CDR-SB*; Clinical dementia rating-sum of boxes score, *APC*; Annualized percent change, *SE*; Standard error of the mean.

We evaluated baseline CSF HFABP levels analyzed using a multiplex-based immunoassay panel. This immunoassay panel, based on Luminex xMAP immunoassay technology and developed by Rules Based Medicine (MyriadMBM), measures a range of lipid, inflammatory, metabolic, and other AD-relevant indices (for further details, please see reference 18). We also examined baseline CSF Aβ_1–42_ and CSF p-tau_181p_ levels, determined using the AlzBio3 Luminex xMAP immunoassay (Innogenetics, Ghent, Belgium). Using previously proposed CSF cutoffs ^19^ we classified participants based on low (<192 pg/ml) and high (>192 pg/ml) Aβ_1–42_ levels and high (>23 pg/ml) and low (<23 pg/ml) p-tau_181p_ levels.

We examined 1205 T_1_-weighted MRI scans. We performed quantitative volume and surface-based analysis of all baseline MRI scans using automated region-of-interest (ROI) labeling techniques [35,36], primarily focusing on the entorhinal cortex, a medial temporal lobe region that is selectively affected in the earliest stages of AD [37] (Figure 1). To additionally investigate neuroanatomic regions that are involved in the later stages of the disease process [37,38], and to minimize multiple comparisons, we averaged longitudinal volume change in the temporal pole, parahippocampal gyrus, inferior temporal gyrus, banks of the superior temporal sulcus, inferior parietal lobule, amygdala, and hippocampus to create an 'AD-vulnerable’ ROI [33,34] (Figure 1). Using an image-analysis method developed within our laboratory [39], we assessed longitudinal sub-regional change in gray matter volume (atrophy) on serial MRI scans (see Additional file 1 for additional details).

For the primary analyses, we used linear mixed effects models to examine the associations of CSF HFABP, CSF Aβ_1–42_ status, and CSF p-tau_181p_ status on atrophy rate of the entorhinal cortex, and of the AD-vulnerable ROI. All analyses co-varied for the effects of the baseline age, sex, presence ("carriers") or absence ("non-carriers") of at least one ϵ4 allele of apolipoprotein E (APOE ϵ4), diagnostic status (AD vs MCI vs HC), and disease severity (CDR-Sum of Boxes score at baseline).

Specifically, our initial model was:

(1) $${\mathrm{\Delta v}=b}_{0}+b_{1}\mathrm{\Delta t}+b_{2}\mathrm{CSF}\_\mathrm{HFABP}\times \mathrm{\Delta t}+\mathrm{covariates}\times \mathrm{\Delta t}+e$$

Here, Δv is the entorhinal cortex or AD-vulnerable ROI thickness change from baseline (millimeters^3^) and Δt is the change in time from baseline MRI scan (years).

Next, investigated whether interactions of CSF HFABP with CSF Aβ_1–42_ status, and CSF p-tau_181p_ status were significantly associated with atrophy over time. Specifically:

(2) $${\mathrm{\Delta v}=\beta }_{0}+\beta_{1}\mathrm{\Delta t}+\beta_{2}\mathrm{CSF}\_\mathrm{HFABP}\times \mathrm{\Delta t}+\beta_{3}{\mathrm{CSF}\_\mathrm{Ab}}_{1–42}\_\mathrm{status}\times \mathrm{\Delta t}+\beta_{4}{\mathrm{CSF}\_p‒\mathrm{tau}}_{181p}\_\mathrm{status}\times \mathrm{\Delta t}+\beta_{5}\left[\mathrm{CSF}\_\mathrm{HFABP}\times {\mathrm{CSF}\_\mathrm{Ab}}_{1–42}\_\mathrm{status}\times \mathrm{\Delta t}\right]+\beta_{6}\left[\mathrm{CSF}\_\mathrm{HFABP}\times {\mathrm{CSFp}‒\mathrm{tau}}_{181p}\_\mathrm{status}\;\times \mathrm{\Delta t}\right]+\mathrm{covariates}\times \mathrm{\Delta t}+e$$

In both models, the main effects of all variables were also included. For brevity, we focus above on the effects of interest.

## Abbreviations

HFABP: Heart fatty acid binding protein; Aβ: Amyloid-β; p-tau181p: Phospho-tau; AD: Alzheimer’s disease; MCI: Mild cognitive impairment; HC: Healthy older controls; AD-vulnerable ROI: Neocortical and subcortical regions affected in the later stages of Alzheimer’s disease.

## Competing interests

Dr. Anders M. Dale is a founder and holds equity in CorTechs Labs, Inc, and also serves on the Scientific Advisory Board. The terms of this arrangement have been reviewed and approved by the University of California, San Diego in accordance with its conflict of interest policies.

Dr. Linda K. McEvoy’s has stock options in CorTechs Labs, Inc.

Dr. James B. Brewer holds stock options in CorTechs Labs, Inc and serves on the advisory board and receives financial support from the Eli Lilly Biomarker Unit (Amyvid). Dr. Brewer also receives research support from General Electric and Janssen Alzheimer Immunotherapy.

Dr. Kaj Blennow has served on the advisory boards for Innogenetics, Lilly, Pfizer and Roche.

## Authors’ contributions

Drs. D, McE, H and D were involved with conception, design, and interpretation of data. Drs. D, McE, H and D provided general overall supervision of the study. Drs. D and T were involved with data analysis. Drs. D, B, McE, A, and D acquired funding. All authors contributed to drafting and critical revision of the manuscript and have given final approval of the version to be published.

## Supplementary Material

Additional file 1 Methods and results. Click here for file
