# Supplementary material for: Heart fatty acid binding protein and Aβ-associated Alzheimer’s neurodegeneration
Source: Mol Neurodegener. 2013 Oct 2;8:39. doi: 10.1186/1750-1326-8-39 (PMC3850652; doi:10.1186/1750-1326-8-39)
Supplement: Additional file 1 — Methods and results. [file 1750-1326-8-39-S1.docx]

**SUPPLEMENTAL METHODS**

**Clinical Assessments**

All participants were selected from the Alzheimer’s Disease Neuroimaging Initiative (ADNI) database (adni.loni.ucla.edu). The ADNI was launched in 2003 by the National Institute on Aging (NIA), the National Institute of Biomedical Imaging and Bioengineering (NIBIB), the Food and Drug Administration (FDA), private pharmaceutical companies and non-profit organizations, as a $60 million, 5-year public-private partnership. The primary goal of ADNI has been to test whether serial magnetic resonance imaging (MRI), positron emission tomography (PET), other biological markers, and clinical and neuropsychological assessment can be combined to measure the progression of mild cognitive impairment (MCI) and early Alzheimer’s disease (AD). Determination of sensitive and specific markers of very early AD progression is intended to aid researchers and clinicians to develop new treatments and monitor their effectiveness, as well as lessen the time and cost of clinical trials. ADNI is the result of efforts of many co-investigators from a broad range of academic institutions and private corporations, and subjects have been recruited from over 50 sites across the U.S. and Canada. The initial goal of ADNI was to recruit 800 subjects but ADNI has been followed by ADNI-GO and ADNI-2. To date these three protocols have recruited over 1500 adults, ages 55 to 90, to participate in the research, consisting of cognitively normal older individuals, people with early or late MCI, and people with early AD. The follow up duration of each group is specified in the protocols for ADNI-1, ADNI-2 and ADNI-GO. Subjects originally recruited for ADNI-1 and ADNI-GO had the option to be followed in ADNI-2. For up-to-date in formation, see www.adni-info.org.

Each participant was formally evaluated using eligibility criteria that are described in detail elsewhere (<http://www.adni-info.org/index.php?option=com_content&task=view&id=9&Itemid=43>). The institutional review boards of all participating institutions approved the procedures for this study. Written informed consent was obtained from all participants or surrogates. Experienced clinicians conducted independent semi-structured interviews with the participant and a knowledgeable collateral source that included a health history, neurological examination, and a comprehensive neuropsychological battery.

We selected participants from the ADNI database if they were clinically diagnosed at baseline as cognitively normal (n = 90), amnestic mild cognitive impairment (MCI) as defined using the revised MCI criteria [1] (n = 139) or probable AD (n = 66).

**MR Image Processing**

All ADNI MRI scans were acquired at multiple sites using either a GE, Siemens, or Philips 1.5T system. Parameter values vary depending on scanning site and can be found at <http://www.loni.ucla.edu/ADNI/Research/Cores/>. Multiple high-resolution T1- weighted volumetric MRI scans were collected for each subject and the raw DICOM images were downloaded from the public ADNI site (<http://www.loni.ucla.edu/ADNI/Data/index.shtml>). All MRI scans were analyzed using a modified version of the FreeSurfer software package (<http://surfer.nmr.mgh.harvard.edu>). These analysis procedures have been applied, validated, and described in detail in a number of publications [2]. In brief, the MRI scans were reviewed for quality, automatically corrected for spatial distortion due to gradient nonlinearity [3], registered and averaged to improve the signal to noise ratio. The cortical surface was automatically reconstructed [4,5] and gray matter thickness measurements were obtained at each point across the cortical mantle [6].

In this study, we primarily focused on the entorhinal cortex because AD-specific pathology is evident in this region in the earliest stages of the disease process [11-13]. To additionally investigate neuroanatomic regions that are involved in the later stages of the disease process [11-14], we averaged longitudinal volume change in the temporal pole, parahippocampal gyrus, inferior temporal gyrus, banks of the superior temporal sulcus, inferior parietal lobule, and hippocampus to create an ‘AD-vulnerable’ region of interest (ROI). All neocortical regions were delineated using an automated, surface-based parcellation atlas [7]. The hippocampus was identified using an automated, subcortical, segmentation atlas [15]. For the analysis of the longitudinal volume change, gray matter thickness change was examined using Quarc (quantitative anatomical regional change), a recently developed method from our laboratory [8,9]. Briefly, each participant's follow-up image was affine-aligned to the baseline scan and locally intensity-normalized. Using nonlinear registration, a deformation field was then calculated to locally register the images with high fidelity for both large- and small-scale structures, including those with low boundary contrast. From the deformation field, a volume-change field (atrophy) can directly be calculated. Using the baseline subcortical and cortical labels, the volume-change field can be sampled at points across the cortical surface or averaged over subcortical regions to give the percent volume change for those ROIs.

**Cerebrospinal Fluid Measures**

Methods for CSF acquisition and biomarker measurement using the ADNI cohort have been reported previously [10]. In brief, CSF was collected and stored at -80°C at the University of Pennsylvania ADNI Biomarker Core Laboratory. Amyloid-β from peptides 1-42 and tau phosphorylated at threonine 181 was measured using the multiplex xMAP Luminex platform (Luminex Corp, Austin TX) with Innogenetics (INNOBIA AlzBio3, Ghent, Belgium) immunoassay kit–based reagents.

Baseline levels of CSF heart fatty acid binding protein (HFABP), apolipoprotein (Apo) C III, Apo D, and Apo E were analyzed using a multiplex-based immunoassay panel. This immunoassay panel, based on Luminex xMAP immunoassay technology and developed by Rules Based Medicine (MyriadMBM), measures a range of lipid, inflammatory, metabolic, and other AD-relevant indices (for further details see reference 16).

**SUPPLEMENTAL RESULTS**

*CSF HFABP, CSF Aβ_1-42_, CSF p-tau_181p_, and longitudinal cognitive decline*

Using the linear mixed effects framework described for longitudinal brain atrophy within the main manuscript, we evaluated the relationship between CSF HFABP x CSF Aβ_1-42_ status x time + CSF HFABP x CSF p-tau_181p_ status x time on change in ADAS-Cog over time among all 295 participants. In these analyses we did not find significant interactions between CSF HFABP, CSF Aβ_1-42_ status and time (β-coefficient = 0.93, SE = 1.2, p-value = 0.42) and CSF HFABP, CSF p-tau_181p_ status and time (β-coefficient = -1.5, SE = 1.2, p-value = 0.22) on change in ADAS-Cog over time. With the interaction terms in the model, only the effect of the CSF p-tau_181p_ status by time was significant (β-coefficient = 1.28, SE = 0.63, p-value = 0.04); the effects of CSF HFABP and CSF Aβ_1-42_ status by time were not significant. Neither any of the main effects nor any of the co-variate effects by time were significant.

**SUPPLEMENTAL REFERENCES**

1. Petersen RC. [Mild cognitive impairment as a diagnostic entity.](http://www.ncbi.nlm.nih.gov.ezp-prod1.hul.harvard.edu/pubmed/15324362) *J Intern Med*. 2004;256:183-94.
2. Fennema-Notestine C, Hagler DJ Jr, McEvoy LK, Fleisher AS, Wu EH, Karow DS, Dale AM; Alzheimer's Disease Neuroimaging Initiative. [Structural MRI biomarkers for preclinical and mild Alzheimer's disease.](http://www.ncbi.nlm.nih.gov.ezp-prod1.hul.harvard.edu/pubmed/19277975) *Hum Brain Mapp*. 2009;30:3238-53.
3. Jovicich J, Czanner S, Greve D, Haley E, van der Kouwe A, Gollub R, Kennedy D, Schmitt F, Brown G, Macfall J, Fischl B, Dale A. [Reliability in multi-site structural MRI studies: effects of gradient non-linearity correction on phantom and human data.](http://www.ncbi.nlm.nih.gov/pubmed/16300968) *Neuroimage*. 2006;30:436-43.
4. Dale AM, Fischl B, Sereno MI. [Cortical surface-based analysis. I. Segmentation and surface reconstruction.](http://www.ncbi.nlm.nih.gov/pubmed/9931268) *Neuroimage*. 1999;9:179-94.
5. Fischl B, Sereno MI, Dale AM. [Cortical surface-based analysis. II: Inflation, flattening, and a surface-based coordinate system.](http://www.ncbi.nlm.nih.gov.ezp-prod1.hul.harvard.edu/pubmed/9931269) *Neuroimage*. 1999;9:195-207.
6. Fischl B, Dale AM. [Measuring the thickness of the human cerebral cortex from magnetic resonance images.](http://www.ncbi.nlm.nih.gov/pubmed/10984517) *Proc Natl Acad Sci*. 2000;97:11050-5.
7. Desikan RS, Ségonne F, Fischl B, Quinn BT, Dickerson BC, Blacker D, Buckner RL, Dale AM, Maguire RP, Hyman BT, Albert MS, Killiany RJ. An automated labeling system for subdividing the human cerebral cortex on MRI scans into gyral based regions of interest. *Neuroimage*, 2006;31: 968-80.
8. Holland D, Brewer JB, Hagler DJ, Fenema-Notestine C, Dale AM. [Subregional neuroanatomical change as a biomarker for Alzheimer's disease.](http://www.ncbi.nlm.nih.gov.ezp-prod1.hul.harvard.edu/pubmed/19996185) *Proc Natl Acad Sci.* 2009;106:20954-20959.
9. Holland D and Dale AM. Nonlinear registration of longitudinal images and measurement of change in regions of interest. *Medical Image Analysis*, in press.
10. Shaw LM, Vanderstichele H, Knapik-Czajka M, Clark CM, Aisen PS, Petersen RC, Blennow K, Soares H, Simon A, Lewczuk P, Dean R, Siemers E, Potter W, Lee VM, Trojanowski JQ; Alzheimer's Disease Neuroimaging Initiative. [Cerebrospinal fluid biomarker signature in Alzheimer's disease neuroimaging initiative subjects.](http://www.ncbi.nlm.nih.gov.ezp-prod1.hul.harvard.edu/pubmed/19296504) *Ann Neurol*. 2009;65:403-13.
11. Braak H, Braak E. [Neuropathological stageing of Alzheimer-related changes.](http://www.ncbi.nlm.nih.gov.ezp-prod1.hul.harvard.edu/pubmed/1759558) *Acta Neuropathol*. 1991;82:239-59.
12. Arriagada PV, Growdon JH, Hedley-Whyte ET, Hyman BT. [Neurofibrillary tangles but not senile plaques parallel duration and severity of Alzheimer's disease.](http://www.ncbi.nlm.nih.gov.ezp-prod1.hul.harvard.edu/pubmed/1549228) *Neurology*. 1992;42:631-9.
13. Gómez-Isla T, Price JL, McKeel DW Jr, Morris JC, Growdon JH, Hyman BT. [Profound loss of layer II entorhinal cortex neurons occurs in very mild Alzheimer's disease.](http://www.ncbi.nlm.nih.gov.ezp-prod1.hul.harvard.edu/pubmed/8699259) *J Neurosci*. 1996;16:4491-500.
14. Arnold SE, Hyman BT, Flory J, Damasio AR, Van Hoesen GW. [The topographical and neuroanatomical distribution of neurofibrillary tangles and neuritic plaques in the cerebral cortex of patients with Alzheimer's disease.](http://www.ncbi.nlm.nih.gov.ezp-prod1.hul.harvard.edu/pubmed/1822725) *Cereb Cortex*. 1991;1:103-16.
15. Fischl B, Salat DH, Busa E, Albert M, Dieterich M, Haselgrove C, van der Kouwe A, Killiany R, Kennedy D, Klaveness S, Montillo A, Makris N, Rosen B, Dale AM. [Whole brain segmentation: automated labeling of neuroanatomical structures in the human brain.](http://www.ncbi.nlm.nih.gov.ezp-prod1.hul.harvard.edu/pubmed/11832223) *Neuron*. 2002;33:341-55.
16. Biomarkers Consortium Data Primer – ADNI, Version December 28, 2011.
